# The evolution of social networks through the implementation of evidence-informed decision-making interventions: a longitudinal analysis of three public health units in Canada

**DOI:** 10.1186/s13012-015-0355-5

**Published:** 2015-12-03

**Authors:** Reza Yousefi-Nooraie, Maureen Dobbins, Alexandra Marin, Robert Hanneman, Lynne Lohfeld

**Affiliations:** Health Research Methodology Program, Faculty of Health Sciences, McMaster University, Hamilton, Canada; School of Nursing and Department of Clinical Epidemiology and Biostatistics, McMaster University, Hamilton, Canada; Department of Sociology, University of Toronto, Toronto, Canada; Department of Sociology, College of Humanities, Arts, and Social Sciences, University of California, Riverside, USA; Department of Clinical Epidemiology and Biostatistics, McMaster University, Hamilton, Canada; 175 Longwood Road South, Suite 210a, Hamilton, ON L8P 0A1 Canada

**Keywords:** Evidence-informed decision-making, Social network analysis, Stochastic actor-oriented modeling, Social selection, Longitudinal analysis

## Abstract

**Background:**

We studied the evolution of information-seeking networks over a 2-year period during which an organization-wide intervention was implemented to promote evidence-informed decision-making (EIDM) in three public health units in Ontario, Canada. We tested whether engagement of staff in the intervention and their EIDM behavior were associated with being chosen as information source and how the trend of inter-divisional communications and the dominance of experts evolved over time.

**Methods:**

Local managers at each health unit selected a group of staff to get engage in Knowledge Broker-led workshops and development of evidence summaries to address local public health problems. The staff were invited to answer three online surveys (at baseline and two annual follow-ups) including name generator questions eliciting the list of the staff they would turn to for help integrating research evidence into practice. We used stochastic actor-oriented modeling to study the evolution of networks. We tested the effect of engagement in the intervention, EIDM behavior scores, organizational divisions, and structural dynamics of social networks on the tendency of staff to select information sources, and the change in its trend between year 1 and year 2 of follow-up.

**Results:**

In all the three health units, and especially in the two units with higher levels of engagement in the intervention, the network evolved towards a more centralized structure, with an increasing significance of already central staff. The staff showed greater tendencies to seek information from peers with higher EIDM behavior scores. In the public health unit that had highest engagement and stronger leadership support, the engaged staff became more central. In all public health units, the engaged staff showed an increasing tendency towards forming clusters. The staff in the three public health units showed a tendency towards limiting their connections within their divisions.

**Conclusions:**

The longitudinal analysis provided us with a means to study the microstructural changes in public health units, clues to the sustainability of the implementation. The hierarchical transformation of networks towards experts and formation of clusters among staff who were engaged in the intervention show how implementing organizational interventions to promote EIDM may affect the knowledge flow and distribution in health care communities, which may lead to unanticipated consequences.

**Electronic supplementary material:**

The online version of this article (doi:10.1186/s13012-015-0355-5) contains supplementary material, which is available to authorized users.

## Background

Organizational innovations can affect social relations within networks. Implementation of innovations is a complex and dynamic process by which the people who are bound within relationships with each other in a social context make adjustments to achieve desired outcomes [1, 2]. Changes in personal and collective knowledge and attitudes over time may affect individuals’ choices for interaction, and subsequently affect network composition [3].

Interventions to promote evidence-informed decision-making (EIDM), like other organizational behavior change interventions, may have social consequences and affect how individuals interact with each other [3]. While implementation frameworks highlight the role of contextual and social factors [4], many identify them as barriers/facilitators of the process of EIDM and not the outcomes that are influenced by it [5–7]. We do not know much about how implementation of EIDM interventions affects the social structure of health care settings.

In a recent 2-year study, we examined the information-seeking relationships of staff of three public health units in Ontario, Canada, before and after implementing a multi-faceted and site-tailored EIDM intervention. Our goal was to understand how information-seeking networks evolved over time and how engagement in the intervention and evidence-informed behavior of staff associated with their evolving network positions and relational tendencies.

### An organizational intervention to promote EIDM in public health organizations

Three public health units in Ontario, Canada, participated in a 22-month multi-faceted and site-tailored intervention to promote EIDM among public health professionals [8]. Senior management from each health unit helped in tailoring the intervention to their unit’s goals for EIDM and available resources. The intervention consisted of an introductory workshop introducing the study and the concept of EIDM, and face-to-face mentoring of small groups of staff through the EIDM process by a professional knowledge broker (KB) [9]. More details about the capabilities and responsibilities of the KB are provided elsewhere [10].

In each public health unit, a group of staff was recruited by local managers to get engaged in the development of summary evidence reviews to address local public health problems, while the majority of their peers had very limited contact with the intervention. Local managers chose these individuals because their roles were already (or were planning to be) associated with EIDM. The KB interacted with this “highly engaged” staff either one-on-one (through consultations) or as members of project-specific teams to develop summary evidence reviews. During and after the intervention, “highly engaged” staff continually communicated with their peers, through which they had the opportunity to share their experience and accomplishment and get recognized by the staff as EIDM experts.

### The evolution of information-seeking networks

Little is known about the effect of organizational interventions on the dynamics of social relations among the members of the organization. Garcia [3] proposed that large group interventions that engage the whole organization in the change process would affect the social networks in three ways: they establish new information-sharing relations that span structural holes (that separate social clusters with limited communication with each other); they form strong ties (based on trust and frequent communications) among participants; and they provide the participants with a broader system-level identification, resulting in more frequent inter-unit communications. Gesell et al. [11] used a network diagnostic tool consisting of structural indicators of cohesion to test if the implementation of a group intervention would increase the network cohesion and connectivity as a result of frequent interactions among participants and found an increase in the indicators after intervention.

**Fig. 1:**
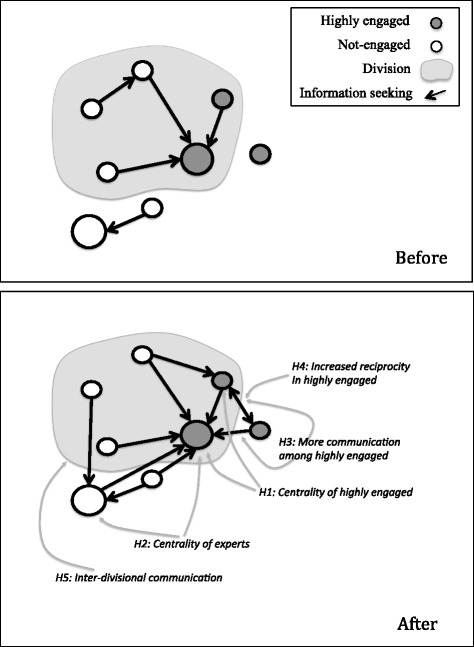
The hypothetical diagram of the expected changes in social relations as a result of the organizational intervention

We expected that the intervention would affect the tendency of staff to seek information from experts in EIDM, the distribution of knowledge in the organization, the tendency of highly engaged staff to communicate with each other, and inter-divisional communication. Figure 1 summarized the hypothetical effects of the intervention, as explained in more details in the following sections.

### Turning to experts

In organizational interventions, to promote EIDM, it is often more justifiable to train a selected group of health practitioners, rather than educating all the organization. “Highly engaged” staff were mainly chosen based on the relevance of their formal roles to EIDM. Especially in health units A and C, some of them had managerial roles with the power to promote the implementation of the intervention. And, especially in unit A, many of them were already at the center of information-seeking networks and were recognized by many in the health unit as experts in EIDM [12, 13]. This selected group could be considered as “lead users” of the innovation [14]. They were supposed to apply expertise gained through their training to assist other staff to find, appraise, and apply research evidence in their practice.

So, we hypothesized that the highly engaged subgroup would gradually take a more central position in the social network over time, as noted below:

*Hypothesis H1: The tendency of staff to seek information from highly engaged peers would increase over time*

In addition to her interactions with highly engaged group, the KB also communicated with the senior management of each health unit in the development of organizational policies related to EIDM. As a result of this, and potential social influence effect of highly engaged members, we expected that the intervention would affect the attitude of the staff towards the relevance of EIDM to their practice and their engagement in EIDM activities. So, they would be more likely to consider the expertise in EIDM when choosing the information sources:

*Hypothesis H2: The tendency of staff to seek information from experts in EIDM would increase over time*

Consequently, selective training of certain staff in the health unit might result in wider recognition of highly engaged staff as experts, thereby leading to an ever-increasing centrality of experts.

### Communication among the highly engaged

The highly engaged group communicated with each other frequently through the development of summary evidence reviews. We anticipated this might result in stronger social ties and bonds of trust among the highly engaged group [3]. So we hypothesized that

*Hypothesis H3: The tendency of highly engaged staff to seek information from each other would increase over time*.

As highly engaged staff feel more confident to engage in conversations regarding to EIDM, we expected to see more reciprocated interaction among a growing number of highly engaged peers (i.e., if A turns to B, B also turns to A for information) [3, 11]. So we hypothesized that

*Hypothesis H4: The tendency of highly engaged staff to reciprocate information-seeking connections would increase over time.*

### Inter-divisional communication

Accessibility of the information source is another contributing factor in shaping information-seeking behaviors, which is represented in the geographical distance, timeliness of the advice, and the level of engagement the advice seeker expects [15].

Peers in the same organizational divisions represent more of these characteristics. People prefer to turn to socially close peers who are geographically accessible and have more common interests and assignments. Empirical evidence in various contexts showed that health practitioners tend to form small local circles based on overlap in professional duties [16–19].

We expected the intervention in this study would promote inter-divisional communications. Co-participation of staff from various divisions in training programs would facilitate inter-divisional communication [3, 11]. In addition, the increasing centrality and recognition of experts would result in more inter-divisional communications towards central experts. Consequently, we tested the following hypothesis:

*Hypothesis H5: The tendency of staff to seek information from peers in other organizational divisions would increase over time.*

## Methods

The three public health units enrolled in the study differed in terms of demographics, organizational structure, and their capacities and policies to promote EIDM. Unit A served a large urban population (>1.5 million). The leaders of the organization were strong advocates of EIDM. At the time the study commenced, unit A had in place many trained project specialists assigned to practice-based teams, with responsibility for conducting literature reviews to address practice issues. Also, more than 100 staff members, mainly managers and project specialists, had attended a weeklong workshop on EIDM. The “highly engaged” staff frequently met at progress meetings and critical appraisal clubs to share their problems and progress with other review teams. At the end of the project, completed reviews were presented in department-wide research events and other local meetings. Based on the KB’s journals, attendance lists of educational workshops and summary evidence review teams, and data exported from the online survey, we classified 51 (8 %) of 620 staff at unit A as highly engaged in the intervention.

Unit B was the largest health unit in the study, serving a large urban population area (>1.5 million). They attached health promotion consultants to specific teams to conduct literature reviews to address practice issues. The adoption of the EIDM intervention was more localized to specific organizational divisions in this unit, within which, managers identified important public health questions and assigned relevant health promotion consultants and other staff to conduct summary evidence reviews. Thirteen staff members (1.2 % of 1068) were highly engaged in the intervention.

Unit C served a smaller mixed urban-rural community (~600,000 population). At unit C, public health nurses had the responsibility for searching and applying evidence to practice, along with carrying out their daily public health duties under the supervision of program managers. Much similar to unit B, a few divisions of unit C participated in the intervention, and nurses were assigned to small groups to conduct summary evidence reviews. There were 18 highly engaged staff members (9 % of 202).

### Data collection

The staff of three health units were invited by senior management to participate in an online survey at baseline and two follow-up assessments with yearly intervals (halfway through the intervention and at the end of the intervention). Participation in the study however was voluntary and confidential. The study was approved by the Hamilton Integrated Review Board (HiREB) and corresponding bodies of three health units.

The staff who consented to participate in the survey answered name generator questions about their information-seeking relationships in the health unit [13]. Respondents named peers to whom they regularly turned to for help integrating research evidence into practice-based decisions.

We used the evidence-based practice (EBP) implementation scale of Mazurek Melnyk and colleagues to assess the extent to which respondents implemented EIDM in their practice [20]. This scale has good internal consistency (Chronbach’s alpha >0.9) and a significant association with educational level and prior exposure to EIDM [20]. It includes 18 items about specific behaviors related to EIDM. Each respondent provided the frequency of his or her involvement in an activity during the 8 weeks prior to survey administration using a five-point frequency scale. The questionnaire was administered at baseline, halfway through the intervention and post-intervention. Non-respondents received two reminder emails 1 week apart [21].

### Descriptive analysis

In the subgroup of participants who provided data at all three time points, we calculated basic aggregate network structural indicators at baseline and the two follow-up assessments, using UCINET 6 [22]. We calculated measures of network connectivity (density, reciprocity, E-I index, and Krackhardt’s hierarchy index) and the positional advantage of network members (in-degree centrality of actors and Freeman’s centralization). The list of measures and their corresponding definitions is provided in the Additional file 1.

### Stochastic actor-oriented models for network evolution

We used stochastic actor-oriented modeling (SAOM) to assess the dynamics of social relations [23]. These models predict the formation of ties among people as the product of various microstructural properties of networks and personal attributes of the people, controlling for internal tendencies of social networks [24]. The main advantage of this approach over conventional regression models is their ability to realistically predict structural tendencies in social networks and consider longitudinal changes as continuous processes rather than discrete changes, while addressing the dependence of observations in social network data [25]. More explanations regarding the specifications and advantages of SAOM models are provided in the Additional file 1.

The included variables, their definitions, and their corresponding hypotheses are provided in Table 1. We included a few structural tendencies as elementary effects in the model, including reciprocity, transitivity, 3-cycle formation, and preferential in-degree centrality (Additional file 1), to assess the dynamics of changes in networks towards a more or less centralized structure.

**Table 1:** Variables included in the stochastic actor-oriented models, their definitions, and corresponding hypotheses

Actor effects
Seeker-highly engaged: The tendency of highly engaged staff to make or maintain ties with others.
Source-highly engaged: The tendency of staff to make or maintain ties with highly engaged staff. Positive changes support hypothesis H1.
Seeker x source-highly engaged: The tendency of highly engaged staff to make or maintain ties with each other. Positive changes support hypothesis H3.
Seeker x source-highly engaged reciprocity: The tendency of highly engaged staff to reciprocate each other’s ties. Positive changes support hypothesis H4.
Seeker-baseline EBP score: The tendency of the staff with higher EBP implementation score to make or maintain ties with others
Source-baseline EBP score: The tendency of staff to make or maintain ties with others with higher baseline EBP implementation score. Positive changes support hypothesis H2.
Seeker-EBP score change: The tendency of the staff with larger improvement in EBP implementation score to make or maintain ties with others.
Source-EBP score change: The tendency of staff to make or maintain ties with others with larger improvement in EBP implementation score. Positive changes support hypothesis H2.
Dyadic effects
Inter-divisional: The tendency of staff to seek information form staff from other divisions. Positive changes supported hypothesis H5.
Structural effects
Reciprocity: The number of reciprocated ties for each actor.
Transitive triplets: The number of transitive patterns in actor A’s connections, which is the number of B,C pairs which actor A is connected to both and also B is connected to C.
3-cycles: A generalized measure of reciprocity. The number of 3-cycles in actor A’s connections, which is the number of B,C pairs which A connects to B, B connects to C, and C connects to A. A negative value for 3-cycle effect along with a positive transitivity effect is an indicator of tendency towards forming local hierarchy.
Preferential in-degree centrality: sum of the in-degrees to actors to whom actor A is connected (the centrality of alter effect), which shows the tendency of network towards centralization.

We included variables corresponding to being highly engaged in the intervention (yes/no), EBP implementation scores at baseline, and the difference of EBP implementation score at follow-up 2 from baseline assessment. We tested hypothesis H1 by analyzing the tendency towards connecting to highly engaged peers and hypothesis H2, the tendency towards connecting to people based on their EIDM behavior. We used the tendency of highly engaged staff to make or maintain ties with each other to test the hypothesis H3. We tested hypothesis H4 about an increased tendency towards reciprocation of information-seeking ties among highly engaged staff by assessing the trend of changes in the reciprocity in the highly engaged group. The tendency towards forming ties with people in other divisions was used to test hypothesis H5.

The value of coefficients for each independent variable in the model is the log odds ratio of the likelihood of actor A making or maintaining connections with actor B vs. actor C who have one unit difference in the independent variable, keeping all other values the same. We included a dummy variable for time, corresponding to the evolution from baseline to follow-up 1 (period 1), and the difference in the parameters between period 2 (follow-up 1 to follow-up 2) and period 1. We used the parameters at period 1 and the changes in parameters from period 1 to period 2 to test the study hypotheses.

Due to basic differences in the structure of the health units in this study and the way the intervention was implemented at each site, we ran SAOM models for each health unit separately. The models were developed in Siena software (version 4.0) in R environment [26].

## Results

At unit A, 119 staff (19 % of all the staff of the health unit) provided the information-seeking network data at all three rounds of assessment (Table 2). The most frequent job titles they held were public health nurse (25 %), supervisor (22 %), consultant or project specialist (16 %), and manager (16 %). Among those, 41 (34 %) were highly engaged in the intervention, half of whom had Masters + educational degree, 14 (34 %) were project specialists, and 11 (27 %) were managers. The highly engaged group had significantly higher EBP scores at baseline, compared to non-involved group (an average score of 12 vs. 9).

**Table 2:** The characteristics of respondents at each health unit based on the availability of network data at baseline and two follow-ups

	Unit A		Unit B		Unit C	
Availability of network data at three time points	Yes^a^	No^b^	Yes^a^	No^b^	Yes^a^	No^b^
Size	119	197	133	401	49	136
Female (%)	111 (93 %)	171 (87 %)	118 (89 %)	364 (91 %)	42 (86 %)	108 (79 %)
Educational degree						
Baccalaureate (%)	71 (60 %)	113 (58 %)	54 (41 %)	208 (52 %)	30 (61 %)	77 (57 %)
Masters+ (%)	42 (35 %)	48 (24 %)	70 (53 %)	122 (31 %)	13 (27 %)	8 (6 %)
Job title						
Manager (%)	19 (16 %)	6 (3 %)	30 (23 %)	28 (7 %)	10 (20 %)	10 (7 %)
Consultant (%)	19 (16 %)	28 (14 %)	37 (28 %)	61 (15 %)	–	–
Nurse (%)	30 (25 %)	71 (36 %)	27 (21 %)	168 (42 %)	24 (49 %)	60 (45 %)
Average years of experience in public health (SD)	13 (8)	8 (8)	17 (9)	13 (9)	13 (9)	12 (9)
EBP score baseline (SD)	11 (7)	10 (10)	10 (9)	10 (10)	8 (7)	7 (7)
EBP score follow-up 2 (SD)	12 (8)	9 (9)	11 (9)	10 (11)	10 (9)	7 (8)
Highly engaged in intervention (%)	41 (34 %)	12 (6 %)	10 (8 %)	3 (0.75 %)	15 (31 %)	3 (2 %)

^a^the staff who provided information for the development of information-seeking network at three time points

^b^the staff who participated in the online survey but either did not answer to the network question or did not participate in three time points

At unit B, 133 staff (12 % of 1068 staff of participating organizational divisions) provided information-seeking data at all three rounds. The most frequent job titles held among the respondents in unit B were consultants (28 %), public health nurses (21 %), and managers (23 %). Of these respondents, 10 (8 %) staff members were highly engaged, of whom 70 % had Masters + degrees, and 70 % were health promotion consultants. Their average baseline EBP score did not differ from the rest of the respondents (10 vs. 10).

At unit C, 49 staff (24 % of the 202 staff of the health unit) included in information-seeking networks at three rounds. The frequently reported job titles were nurse (49 %), public health inspector (14 %), and manager (20 %). In this unit, 15 (31 %) staff members were highly engaged, of whom 25 % had Masters + degrees, 6 (40 %) were nurses, and 4 (27 %) were managers. Their average baseline EBP score (9.6) was higher than the rest of respondents (7.7), but the difference was not statistically significant.

As shown in Table 2, in all the three health units, the staff who provided network data at all time points on average were more educated and more experienced, more likely to be managers and to be highly engaged in the EIDM intervention, compared to other respondents who either did not provide network information in all time points or only answered the EBP questionnaire. This shows that the network analysis is biased towards a subgroup of staff in the health units who were closer to managerial levels and also considered EIDM more relevant to their practice.

### Descriptive analysis of networks

Table 3 shows the aggregate structural indicators of networks at each time point. In unit A, the density of information-seeking networks showed a small increase, while the two other units stayed unchanged. The reciprocity of connections did not change noticeably in unit A, but showed a decrease in unit B, and a transient increase at first follow-up in unit C. In-degree centralization in the three health units showed a transient decrease at first follow-up, which was more prominent in unit C. Krackhardt’s hierarchy index showed a transient decrease towards less hierarchical structure at follow-up 1 in the three units, followed by a subsequent increase in follow-up 2. The E-I index of the three health units was negative, indicating an overall tendency towards intra-divisional connections, but showed a transient decrease (towards less inter-divisional connections) at follow-up 1 in units A and C, and a transient increase (towards more inter-divisional connections) at follow-up 1 in unit B.

**Table 3:** Structural indicators of information-seeking networks in each health unit, at baseline and follow-ups

	Unit A	Unit B	Unit C
Size	119	133	49
Density baseline	1.5 %	1.0 %	3.3 %
Density follow-up 1	1.6 %	0.9 %	3.1 %
Density follow-up 2	2.0 %	1.0 %	3.3 %
Reciprocity baseline	12 %	25 %	16 %
Reciprocity follow-up 1	15 %	22 %	24 %
Reciprocity follow-up 2	13 %	17 %	13 %
In-degree centralization baseline	16 %	5 %	20 %
In-degree centralization follow-up 1	12 %	3 %	14 %
In-degree centralization follow-up 2	17 %	6 %	22 %
Krackhardt’s hierarchy baseline	0.93	0.84	0.92
Krackhardt’s hierarchy follow-up 1	0.86	0.83	0.85
Krackhardt’s hierarchy follow-up 2	0.90	0.95	0.89
Normalized group centrality of highly engaged staff-baseline	0.37	0.11	0.44
Normalized group centrality of highly engaged staff follow-up 1	0.45	0.14	0.35
Normalized group centrality of highly engaged staff follow-up 2	0.56	0.10	0.35
Divisions: E-I index baseline	−0.56	−0.60	−0.34
Divisions: E-I index follow-up 1	−0.60	−0.41	−0.46
Divisions: E-I index follow-up 2	−0.56	−0.45	−0.32

At baseline, 37 % of non-engaged staff of unit A, 11 % of unit B, and 44 % of unit C turned to highly engaged staff for information (group centrality in Table 3). The centrality of the highly engaged group only increased in unit A over time (with an increase to 56 % at follow-up 2).

### Stochastic actor-oriented models

After 2000 iterations in phase 3 of the procedure [26], all three models converged acceptably, with the t-ratios <0.05 for deviations from observed values. The parameter estimates (and standard errors) and their statistical significance in the model are provided in Table 4.

**Table 4:** The log odds ratios (and standard errors) of the effect of personal, dyadic, and structural variables on the likelihood of forming or maintaining information-seeking ties over time in the stochastic actor-oriented models

	Unit A	Unit B	Unit C
Rate parameter-period 1	5.00 (0.54)	3.29 (0.38)	3.38 (0.54)
Rate parameter-period 2	4.54 (0.43)	3.72 (0.44)	2.97 (0.50)
Out-degree (density)-period 1	−3.41 (0.10)*	−3.75 (0.16)*	−3.70 (0.29)*
Out-degree (density)-period 2 change	−0.22 (0.21)	−0.29 (0.30)	0.46 (0.58)
Reciprocity-period 1	1.35 (0.16)*	1.82 (0.19)*	1.30 (0.40)*
Reciprocity-period 2 change	−0.03 (0.32)	−0.61 (0.37)	−0.98 (0.78)
transitive triplets-period 1	0.43 (0.06)*	0.64 (0.15)*	0.93 (0.18)*
transitive triplets-period 2 change	0.26 (0.12)*	0.11 (0.31)	−0.56 (0.37)
3-cycles-period 1	−0.43 (0.13)*	−0.32 (0.28)	−0.44 (0.39)
3-cycles-period 2 change	−0.53 (0.26)*	0.62 (0.58)	−0.67 (0.77)
In-degree-centrality-period 1	0.03 (0.01)*	0.08 (0.04)*	0.18 (0.05)*
In-degree-centrality-period 2 change	0.04 (0.03)	0.14 (0.08)	0.09 (0.11)
Inter-divisional-period 1	−1.63 (0.12)*	−1.36 (0.14)*	−1.31 (0.29)*
Inter-divisional-period 2 change	−0.04 (0.24)	−0.39 (0.26)	0.80 (0.57)
Source-highly engaged-period 1	0.33 (0.10)*	0.34 (0.19)	−0.01 (0.22)
Source-highly engaged-period 2 change	0.002 (0.20)	−0.54 (0.39)	−1.11 (0.45)*
Seeker-highly engaged-period 1	0.16 (0.12)	0.38 (0.27)	0.51 (0.25)*
Seeker-highly engaged-period 2 change	−0.19 (0.24)	0.88 (0.52)	−0.69 (0.52)
Seeker x source-highly engaged-period 1	0.70 (0.22)*	0.90 (0.50)	0.86 (0.47)
Seeker x source-highly engaged-period 2 change	0.39 (0.47)	0.06 (1.05)	1.60 (0.92)
Seeker x source-highly engaged reciprocity-period 1	−0.83 (0.52)	−0.02 (1.68)	0.49 (1.19)
Seeker x source-highly engaged reciprocity-period 2 change	−1.25 (1.05)	4.76 (3.28)	1.74 (2.47)
Source-baseline EBP score-period 1	0.05 (0.007)*	0.02 (0.007)*	0.01 (0.02)
Source-baseline EBP score-period 2 change	0.02 (0.01)*	0.0005 (0.01)	0.05 (0.03)
Seeker-EBP score-period 1	0.0001 (0.008)	0.02 (0.008)*	−0.01 (0.02)
Seeker-EBP score-period 2 change	0.004 (0.02)	0.004 (0.02)	−0.04 (0.04)
Source-EBP score change-period 1	0.02 (0.007)*	0.02 (0.008)*	0.03 (0.01)*
Source-EBP score change-period 2 change	0.007 (0.01)	0.004 (0.02)	0.003 (0.02)
Seeker-EBP score change-period 1	0.01 (0.009)	0.02 (0.01)*	0.003 (0.02)
Seeker-EBP score change-period 2 change	0.03 (0.02)	0.0008 (0.02)	0.07 (0.04)

*p value less than 0.05 for the difference from zero

Only in unit A did the staff show a significant positive tendency to make or maintain ties with highly engaged staff at period 1 (*source-highly engaged*: 0.33), with a small decrease at period 2, compared to a large decrease in tendency at period 2 in the two other units (most prominent in unit C with 1.11 decrease from period 1). These findings are consistent with a rising trend of group centrality of highly engaged staff (Table 3) and support hypothesis H1 in unit A about the tendency of the staff to form or maintain ties with highly engaged peers over time.

At period 1, the staff had a positive tendency to make or maintain ties with others with higher baseline EBP implementation scores (*source-baseline EBP score*), which was statistically significant in units A and B. At period 2, the change in the trend was larger in units C (0.05 increase) and A (0.02 increase), implying an increasing tendency to seek information from staff with higher EBP scores over time. We also observed a positive tendency in staff to make or maintain ties with others with higher improvement in their EBP implementation scores (*source-EBP score change*) at period 1, which was significant in all the three health units. The change at period 2 was small and positive in three units. The positive tendency towards staff with higher EBP scores at period 1 could be explained either by the effect of intervention on the social selection of staff or the already central position of those staff at baseline (perhaps because of their formal roles).

At period 1, the staff with higher baseline EBP scores (seeker-baseline EBP score) and higher changes in scores (seeker-EBP score change) were more active at unit B compared to the other two units. At period 2, the staff with higher changes in EBP scores significantly improved their tendency towards being more active in unit C (0.07) and A (0.03).

In all three health units, highly engaged staff showed a positive tendency to make or maintain ties with each other at period 1 (seeker x source-highly engaged). This tendency increased in period 2, especially in units A and C. These findings imply that the highly engaged staff showed an increasing tendency towards forming clusters with themselves over time.

Among the highly engaged staff, the tendency towards reciprocation did not differ significantly from the rest of the network at period 1. However, in unit A, the already negative tendency in period 1 (seeker x source-highly engaged reciprocity-period 1 = −0.83) decreased even more in period 2 (seeker x source-highly engaged reciprocity-period 2 change = −1.25), but showed some increase in two other units. None of these effects were statistically significant, not supporting hypothesis H4.

The staff in the three health units did not tend to make or maintain ties with other divisions, as indicated by the significant and negative values for inter-divisional connections at period 1. At period 2, the tendency showed a larger decrease in unit B (−0.39), which was consistent with a decrease in E-I index at follow-up 2 in this unit. The tendency only increased in unit C with a 0.80 increase in the coefficient, supporting hypothesis H5 about an increase in inter-divisional connections only in unit C (generally from practice-based divisions towards the supervisory/administrative division). These findings were consistent with a larger increase in E-I index from follow-up 1 to follow-up 2 in unit C, compared to the other two units.

At period 1, the staff in the three health units showed a significant positive tendency to make or maintain reciprocated ties, which was larger in unit B (*reciprocity-period 1*: 1.82). The tendency towards reciprocation decreased at period 2 in all three health units, with a larger decrease in unit C (*reciprocity-period 2 change: −*0.98). The positive reciprocity effect at period 1 shows an internal tendency towards reciprocation in networks, as well as the possibility of a transient increase in tendency towards reciprocation, which was followed by a decrease in tendency. It was also consistent with the observed decrease in reciprocity in all three units from follow-up 1 to follow-up 2 (Table 3).

The preferential in-degree centrality effect, that shows the tendency of actors to make or maintain ties with the staff who are already central, was significant and positive in all networks at period 1, with a larger value in unit C (*in-degree-centrality-period 1:* 0.18). The tendency increased at period 2 in all the three health units.

At period 1, the coefficients for transitivity were significant and positive, and the coefficients for making or maintaining 3-cycles were negative, indicating a tendency to form hierarchical relations. At period 2, the change in trend towards a local hierarchical structure was more prominent in unit A with an increasing transitivity and a decreased tendency towards 3-cycle formation from period 1. At period 2, unit B showed a small increase in transitivity and a large increase in 3-cycle formation (an indicator of generalized reciprocity). These findings in presence of a decreased tendency towards making inter-divisional connections and a decrease in E-I index from follow-up 1 to follow-up 2 imply that the staff tended to maintain and strengthen the intra-divisional ties rather than turning to external experts. In unit C, at period 1, the transitivity effect was highest and 3-cycle effect was lowest among all units (i.e., the largest tendency towards local hierarchy). At period 2, both effects decreased. This finding along with a decreased reciprocity and an increase in tendency of inter-divisional connections implies a trend in staff to make new connections (to the central staff in the supervisory/administrative division) rather than closing triangles. It is also consistent with the observed pattern between follow-up 1 and follow-up 2, which shows more connections from practice-based divisions towards the supervisory/administrative division.

## Discussion

### Centrality of experts

In all three health units, the staff showed larger tendencies to seek information from peers whose behavior was more in line with EIDM principles over time. Only in unit A did the highly engaged staff become more central, supporting hypothesis H1. In all three health units, and especially in units A and C, the network evolved towards a more centralized structure, with a preferential centrality of already central staff.

If the intervention to empower health practitioners through the EIDM process is adopted successfully, we expect that those engaged in the intervention will become increasingly recognized by their peers as experts in EIDM. Analyzing the evolution of networks showed that only in unit A did the highly engaged staff (who were mainly already central at baseline) become even more central over time.

Seeking information from peers in finding and applying research evidence is a routine behavior in public health organizations [13] and among health practitioners [27]. It is rooted in *transactive memory* principles explaining how group members retrieve and distribute knowledge to effectively improve their collective productivity [28]. Group members turn to peers who are considered experts when they need knowledge and expertise that is beyond their personal capabilities. An important requirement for this process of information sharing is that the group members have a tacit knowledge about who knows what in their group [29]. Consequently, it is expected that if an intervention provides opportunities for knowledgeable staff to be presented to and recognized by a larger number of peers, as a result, the knowledgeable staff will be added to the referral directory of more people and will be approached by more over time.

The tendency towards centralization may show that the EIDM intervention was more effective in empowering and popularizing the already known experts, rather than distributing the knowledge more evenly among staff. Bunger et al. observed a similar increase in centralization of clinicians’ advice networks around faculty experts, with a decline of local private connections among clinicians after a learning collaborative intervention, which was the result of a wider recognition of experts by the clinicians [30]. This increased dominance of a small group of experts has both positive and negative consequences. Adoption of innovations is facilitated in centralized networks where there are prestigious actors who have enough credibility to influence the behavior of others and have routes of access to diffuse information to a large group of people [31]. However, strong dependence on a small group of experts may lead to less autonomy and productivity of health practitioners overall. This dominance of a few may hamper the dynamic communication and productivity of staff [17]. In addition, staff may gradually hesitate to turn to those central elite and prefer less optimal local information sources because of their limited availability [15] and larger disparity in social status [32, 33].

The implementers of EIDM training programs should be prepared for such social changes and plan accordingly during the design stage. The implementers should consider whether they aim to train a group of staff as experts in EIDM who will act as information sources and experts or as champions who will enhance the process of organizational change. The former objective would be achieved by choosing the highly engaged group according to their baseline expertise, the relevance of their jobs to EIDM, and also their personal interest in the training. The latter would be fulfilled by choosing individuals at the center of formal and informal social networks and in position of power within the organization.

### Cluster formation

In all units, the highly engaged staff showed an increasing tendency towards forming clusters. This could be the result of the communicative nature of the intervention. The intervention provided the highly engaged staff the opportunity to meet with each other and share their experiences through progress meetings and critical appraisal clubs. Co-participation in those events might have resulted in the formation of sustainable ties.

Formation of ties among staff who are comparable in terms of social and organizational status (so status inequality does not compromise their information-seeking behavior [33]) but are based in different organizational divisions (so they are not bound by the politics and hierarchies of each other’s programs) enhances the voluntary, dynamic, and productive communication, through which the members share their experience and progress, listen to each other’s stories, and provide reflection and feedback [34]. This continued engagement over time may result in development of tacit knowledge and a repertoire of solutions to shared problems that further enhances the solidarity of connections and productivity of communications [35, 36].

Formation of strong cohesive ties among members nurtures the atmosphere of trust, support, and feedback, and is a powerful step in overcoming the social resistance and enhancing the commitment in adopting new innovations [3]. This tendency should coincide with the formation of weaker bridging ties outside the cohesive circle to prevent entrapment of information. Formation of a cohesive core along with continuous development of new weak ties with periphery has been suggested as a successful step in the evolution of networks through organizational innovation processes [37].

These findings imply that the implementers of EIDM interventions should actively consider developing and sustaining social networks as part of the intervention process. This could be achieved by maintaining a communicative training strategy through formation of small work groups and regular progress meetings among the participants, along with promoting informal communications.

### Ties within and between divisions

The staff in the three units showed a tendency towards limiting their connections within their divisions over time. These findings did not support hypothesis H5 about the effect of the intervention in increasing the communication between divisions. The tendency of health practitioners to seek information from socially and geographically proximate peers has been shown in different studies [38, 39]. In addition to the natural tendency to turn to local peers due to ease of access and common values and interests, this tendency may also show an increased autonomy of organizational divisions and reduced reliance on external experts after the intervention.

This finding highlights the need for organizational strategies to maintain and reconnect newly shaped ties by facilitating continuous communication through regular meetings and conventions, developing common programs and tasks, and reducing the cost of maintaining connections by facilitating the formation of third-party linkages or common partners for pairs who bridge clusters [40]. The reactivation of currently inactive bridging ties is less costly than maintaining strong ties and may be even more beneficial than establishing new ties [41].

### Limitations

Low response rate is the main threat to the generalizability of findings of this study. According to our analysis of the characteristics of the non-respondents, our field knowledge, and the results of qualitative interviews with the participants (in progress), many of the non-respondents were the staff who did not consider EIDM relevant to their practice (such as administrative staff). This difference biases the results of the current study to the staff of health units who deal with research evidence more frequently and are more supportive of the EIDM in general, so the conclusions about the effects of the intervention are probably optimistic.

In addition, due to the lack of a parallel control group, our findings on the changes in social networks through the implementation of the intervention could be simply the result of natural tendencies in social networks through time and not the effect of the intervention per se. However, comparison of trends between two periods provides clues for the causality.

## Conclusions

In summary, we found a significant association between engagement in the intervention and improved EIDM behavior with becoming more central in information-seeking networks. The networks became more centralized around a few already central experts, leading to a more hierarchical information-seeking structure. Highly engaged staff formed clusters among themselves. The information could not promote inter-divisional communications. However, where formal EIDM professionals were not available locally (such as in unit C), the staff also turned to known external experts.

An indicator of sustainable organizational change is the extent to which the desired behavior is observed in organizational routines after the innovation was introduced [42]. Our findings showed that social changes continued to occur even after the intervention ended, on outcomes such as the formation of social relations among public health practitioners, centrality of experts, and shaping of clusters. These patterns support the sustainability of the change, especially in one health unit that showed the strongest support by the leaders and participation by the staff. We suggest that longitudinal analysis of professional networks is a helpful tool that can reveal underlying social processes after implementing EIDM interventions if considered as representation of dynamic and complex social processes rather than static and determinate outcomes of intervention [43].

Our findings also confirmed that trajectories of implementing capacity building interventions are complex and far from being a linear domino reaction [44]. Therefore, EIDM implementers should consider and balance different objectives of educational interventions, such as empowering practitioners to deal more independently with research evidence by themselves or facilitating recognition and access to qualified experts who help them through EIDM. In this study, the intervention focused on a subgroup of staff who mainly already held expert and consultant roles. It resulted in an increase in centralization of information-seeking networks around an already well-known group of consultants and managers. On the one hand, this centralization may pave the way to easier access to appropriate experts who can provide high-quality consultations to practice-based teams. On the other hand, the increased status gap may result in an imbalance in the distribution of knowledge in the organization. Further studies are needed to compare the short- and long-term effectiveness of selective training versus broad capacity development on sustainable adoption of EIDM and the distribution of knowledge in public health organizations.

## Additional file

Additional file 1:
**Technical details of descriptive network analysis and stochastic actor-oriented modeling.** (DOC 112 kb)
