# Supplementary material for: The evolution of social networks through the implementation of evidence-informed decision-making interventions: a longitudinal analysis of three public health units in Canada
Source: Implement Sci. 2015 Dec 3;10:166. doi: 10.1186/s13012-015-0355-5 (PMC4669621; doi:10.1186/s13012-015-0355-5)
Supplement: Additional file 1: — Technical details of descriptive network analysis and stochastic actor-oriented modeling. (DOC 112 kb) [file 13012_2015_355_MOESM1_ESM.docx]

**Descriptive analysis of social networks**

We calculated density as a basic measure of connectivity by dividing the number of existing ties by the total possible number of ties among all the network actors. We used reciprocity to quantify the bi-directionality of information-seeking ties [1], which is the percentage of all ties in the network that are reciprocated.

In order to show change in inter-divisional connections, we calculated the E-I index, which is the number of external connections (outside divisions) minus the number of internal connections (within divisions), divided by the number of all connections [1]. The measure ranges from -1 to 1, with negative values indicating a tendency toward intra-divisional and positive values indicating the tendency towards inter-divisional connections.

In-degree centrality (number of connections towards each actor) in directed networks is a rough measure of popularity and prestige [1]. By using the centrality measures of all actors in the network, Freeman’s centralization measures were calculated to give an overall picture of the variation between actors in the network in terms of their centrality. It is the ratio of the sum of the differences of all centralities from the highest centrality in the network over the largest possible sum in networks of the same size. Larger centralization measures indicate more resemblance to a star-shaped network (i.e., if all actors are exclusively connected to one popular actor) [2].

We also measured, Krackhardt’s hierarchy index, which shows the extent to which the paths (including indirect connections between pairs of actors) are not reciprocated; and represents the tendency of the network towards a hierarchical structure [3]. In a perfect connected out-tree (a single stem with all actors located at different levels of the branches all oriented towards the stem) the value of the hierarchy index is 1.

**Advantages of stochastic Actor-oriented model (SAOM):**

Unlike conventional survey data, the relationships of pairs of people in social networks are not independent, and there are structural tendencies in networks that involve more than pairs of actors, which should be considered (or at least adjusted for) in a realistic model. Controlling for structural tendencies is challenging in conventional regression models, which generally assume independence among observations [4].

Observed changes in different time points of longitudinal study of networks do not happen abruptly. They are the result of gradual evolution of network relationships over time [5]. SAOM models assume that observed changes between time points (e.g. baseline and follow up) are the cumulative presentation of several unobserved consecutive micro-steps through which network actors change their ties and re-evaluate the outcomes in an iterative feedback process [6, 7] to optimize their positions in the network, whenever they have the opportunity. Their choices include maintaining a tie with a peer, disconnecting that tie, or making a tie with a new peer [6]. The probability of change at each step depends on the combination of network structure and characteristics of all other actors, and also random error [6].

**Basic structural tendencies in SAOM:**

There are a few structural tendencies of social networks that are strongly suggested to be included in the SAOMs to improve the convergence [8]. They include reciprocity, transitivity, 3-cycle formation, and preferential in-degree centrality.

Reciprocity is a natural tendency of social relations. When actor A does actor B a favor, then actor B will feel the urge to return the favor as a moral obligation (*norm of reciprocity*) [9, 10].

People also tend to close triangles and form social clusters. So if actor A seeks information from actor B, and B turns to C, A has the tendency to also turn to C directly, and form a transitive triplet or triangle. Cartwright and Harary [11] discussed the issue of transitivity as a psychological tendency of humans for balance, which was rooted in Heider’s formulation of balance theory [12]. Alternatively, the above-mentioned triangle between actors A, B, and C may get closed by a connection from actor C to actor A, forming a 3-cycle. A positive effect of transitivity, in presence of a negative effect of 3-cycle formation may show the tendency of network to form local hierarchy [8].

Preferential in-degree centrality indicates the tendency of actors to preferentially attach to already central individuals, which deepens the inequality of actors in the network (rich get richer) [13].

References:

1. Hanneman R, Riddle M: **Introduction to social network methods**. 2005.

2. Freeman L: **Centrality in social networks: Conceptual clarification**. *Soc Networks* 1979, **1**:215–239.

3. Krackhardt D: **Graph Theoretic Dimensions of Informal Organizations**. In *Computational Organizational Theory*. Edited by Carley K, Prietula M. Hillside NJ: Lawrence Erlbaum Assoc; 1994:89–111.

4. Snijders T: **Statistical models for social networks**. *Annu Rev Sociol* 2011, **37**:131–153.

5. Holland P, Leinhardt S: **A Dynamic Model for Social Networks**. *J Math Sociol* 1977, **5**:5–20.

6. Snijders TAB, Van de Bunt GG, Steglich CEG, van der Bunt G: **Introduction to stochastic actor-based models for network dynamics**. *Soc Networks* 2010, **32**:44–60.

7. Snijders T: **Stochastic actor‐oriented models for network change**. *J Math Sociol* 1996, **21**:149–172.

8. Ripley R, Snijders T, Boda Z, Voros A, Preciado P: *Manual for SIENA Version 4.0 (version December 9, 2014)*. Oxford: University of Oxford, Department of Statistics, Nuffield College; 2014.

9. Gouldner A: **The norm of reciprocity: A preliminary statement**. *Am Sociol Rev* 1960:161–178.

10. Wasserman S, Faust K: *Social Network Analysis: Methods and Applications*. Cambridge: Cambridge University Press; 1994.

11. Cartwright D, Harary F: **Structural balance: a generalization of Heider’s theory**. *Psychol Rev* 1956, **63**:277–293.

12. Heider F: **Attitudes and cognitive organization**. *J Psychol* 1946, **21**:107–112.

13. Barabasi A, Albert R, Barabási A: **Emergence of scaling in random networks**. *Science (80- )* 1999, **286**:509–512.
